# The effect of environmental factors and migration dynamics on the prevalence of antibiotic-resistant *Escherichia coli* in estuary environments

**DOI:** 10.1038/s41598-018-20077-x

**Published:** 2018-01-26

**Authors:** Guangshui Na, Zihao Lu, Hui Gao, Linxiao Zhang, Qianwei Li, Ruijing Li, Fan Yang, Chuanlin Huo, Ziwei Yao

**Affiliations:** 1 Key Laboratory for Ecological Environment in Coastal Areas (SOA), National Marine Environmental Monitoring Center, Dalian, 116023 China; 2 School of Biology Technology, Dalian Polytechnic University, Dalian, 116034 China; 3 School of Marine Science, Shanghai Ocean University, Shanghai, 201306 China

## Abstract

Understanding the antibiotic resistance transmission mechanisms and migration dynamics of antibiotic-resistant bacteria (ARB) in the natural environment is critical given the increasing prevalence of antibiotic resistance. The aim of this study was to examine the fate of sulfonamide-resistant fecal bacteria (*E*. *coli*) in an estuary ecosystem and to explore the role and contribution of environmental factors in this process. The prevalence of sulfonamide-resistance status of *E*. *coli* was analyzed over different seasons in two estuary systems. Environmental factors and disturbance indices of anthropogenic activities were evaluated by detecting antibiotic concentrations, heavy metal abundance and other physicochemical parameters. The abundances of antibiotic-resistant *E*. *coli* were significantly attenuated during land-sea migration suggesting that estuary environments play a natural mitigation role in the contamination of freshwaters by antibiotic-resistant *E*. *coli*. Additionally, environmental factors and disturbance indices of anthropogenic activities significantly correlated with the distribution and migration of antibiotic-resistant *E*. *coli* in the estuaries. Lastly, simulation experiments suggested differential adaptability between antibiotic-resistant and non-resistant *E*. *coli* towards environmental changes in estuary environments. Meanwhile, our results indicate that low concentrations of antibiotics will not increase the competitive advantage of resistant *E*. *coli* in estuaries.

## Introduction

The increase in the number of antibiotic-resistant and multi-resistant bacterial strains is a major health security challenge of the twenty-first century and poses a serious threat to clinical practices. At least 700,000 people are killed by antibiotic-resistant bacteria each year, and this figure is expected to exceed 10,000,000 in 2050 according to a World Health Organization report^1^. Further, the scale of the problem is global and not confined to individual nations or regions. Among 17,309 *E*. *coli* clinical collected isolates from 20 Chinese hospitals, the prevalence of Ampicillin resistance was 85.5%^2^. Further, ciprofloxacin-resistant and sulfamethoxazole-resistant strains accounted for an average of 57.8% and 58.1% of *E*. *coli* isolates in the same hospitals, respectively. Likewise, Aminopenicillin resistance has been observed to be very common in Europe, with national resistance ranging from 34.7% to 73.0% in Finland and Bulgaria, respectively, in 2014^1^. Although antibiotic resistance has become a major threat to human health worldwide, this phenomenon has been largely overlooked in environmental studies.

Aquatic environments may provide an ideal setting for the acquisition and dissemination of antibiotic resistance. Antibiotic-resistant bacteria (ARB) may further migrate and interact with natural environmental strains, eventually leading to the development of a natural reservoir for various antibiotic resistance genes (ARGs) in environmental bacteria^3^. As a direct consequence of replicative and propagative biological characteristics of ARGs and ARB, the ecological hazard caused by antibiotic resistance is more directed, prone to spread, and harder to control and eliminate. Multiple varieties of ARB have been recently detected in dairy lagoons, wastewater treatment plants, rivers and marine environments^4–7^. While strengthening proper antibiotic usages and accelerating the development of new drugs are needed to mitigate the spread of antibiotic resistance, understanding the movement of ARB and ARG in environments is also critical. Thus, a more complete understanding of the migration dynamics and transmission mechanisms of antibiotic resistance in natural aquatic environments is extremely important towards reducing the impact of antibiotic resistance and limiting its dispersal.

Estuary ecosystems are ecotones between rivers and oceans, exhibit unique physical and chemical properties and represent the strongest interaction between continental and oceanic environments. Estuaries are also a key area for understanding ARB fate after release into aquatic environments. During the migration of ARB from estuarine to marine environments, the spread of antibiotic resistance may become significantly more difficult with increasing environmental change, due to the inhibition of vertical transmission of ARB^8^. Additionally, adaptation to a specific environment (e.g., the acquisition of ARG in a host) may result in corresponding fitness changes in other environments, potentially resulting in additional environmental filtering of ARB in estuaries^9^. However, limited information is available regarding the population dynamics of ARB after release into estuary environments. In particular, migration patterns of ARB and the influence of environmental change in the retention of ARB in estuary environments is unclear. The present study aimed to examine the distribution and migration dynamics of sulfonamide-resistant *E*. *coli* in two estuaries under differential influence of anthropogenic activities and explore the role and contribution of environmental factors in this process.

We characterized the occurrence and distribution of sulfonamide-resistant *E*. *coli* in summer and winter in the Daliaohe and Liaohe river estuaries. We also assessed comprehensive environmental factors index via three metrics: three types of antibiotics, six typical heavy metals, and environmental physicochemical parameters. Here, we provide evidence for the complex relationships among sulfonamide-resistant *E*. *coli*, antibiotic compounds, heavy metal contaminants, and other environmental factors. Furthermore, this study also provides neoteric perspective to reassess the role and potential risk of environmental pressures from non-antibiotic contaminants on sulfonamide-resistant *E*. *coli* migration.

## Materials and Methods

### Sampling Design

The Liaohe and Daliaohe River water catchments are located between 40° 31ʹ N to 45° 17ʹ N and 116° 54ʹ E to 125° 32ʹ E in northeastern China, which can provide an excellent comparative opportunity to assess the fate of ARB in estuary environments that experience differential anthropogenic influences^8,10^. Surface water samples were collected along the Daliaohe and Liaohe River estuaries during the dry season in April 2014 and the wet season in August 2015. Sample sites were chosen across salinity gradients to encompass the range of spatial environmental heterogeneity. More details are shown in SI Appendix, Figure S1. Water samples were collected at a depth of approximately 10 cm in autoclaved 1.0 L Nalgene bottles. Water temperature, dissolved oxygen (DO), and pH were measured using a WTW Cond 340i SET B hand-held field probe (Table 1). All samples were placed in an ice bath and transported to the laboratory within 6 h after collection. Water samples were stored at 4 °C until processing.

**Table 1:** Water quality measurements in 2015. The water quality measurements in 2014 could be seen in the article published in the 2015^8^.

Daliaohe River estuary			Liaohe River estuary		
Salinity (‰)	T (°C)	DO	Salinity (‰)	T (°C)	DO
27.9	27.5	4.59	28.8	26.4	5.34
25.3	27.8	3.85	28	27.1	4.1
19.8	28	3.51	25.9	26.8	4.49
15.4	28.4	4.41	17.3	28.1	3.34
10	28.7	4.45	9.7	28.3	4.1
6.4	28.9	4.44	6.4	28.3	5.8
—	—	—	1.2	27.9	6.5

### Assessment of Antibiotic Resistance in *E*. *coli* Isolates from Environmental Waters

Firstly, MI Medium was formulated according to the instruction and used to prepare pour plates. Half of the MI agar plates (without antibiotics), the rest as MI-R agar plate with minimum inhibitory concentration of sulfonamide (350 μg mL^−1^). An appropriate water sample volume (i.e., 50 mL, 100 mL, 300 mL) was selected based on the collection area and estimation of pollution. These water samples were filtered through 0.45-μm membrane and the filters transferred onto the prepared MI and MI-R plates, and incubated at 35 °C for 24 h. After incubation, blue colonies were counted under ambient light. *E*. *coli* ATCC 25922 was used as a control strain having no resistance to the selected antibiotics. The abundance of *E*. *coli* was calculated according to the following equation:

1 $$E.coli/\mathrm{100\; mL}=\frac{E.\,coli\,{\rm{abundance}}}{{\rm{sample}}\,{\rm{value}}\,(\mathrm{mL})}\times 100$$

The percent of *E*. *coli* that exhibited antibiotic resistance was calculated using the following equation:

2 $$ \% \,{\rm{Resistance}}=\frac{E.\,coli\,{\rm{on}}\,{\rm{antibiotic}}\,{\rm{plate}}}{E.\,coli\,{\rm{on}}\,{\rm{control}}\,{\rm{plate}}}\times 100$$

### High-performance Liquid Chromatography-Mass Spectrometry (HPLC-MS/MS) Analysis of Antibiotics

Pretreatment of surface water samples and solid-phase extraction procedures were performed to clean the antibiotic samples with an Oasis® MCX cartridge (3 mL, 60 mg) and an Oasis® HLB cartridge (3 mL, 60 mg). The samples were then analyzed using TSQ Quantum high-performance liquid chromatography-mass spectrometry (Thermo Fisher Scientific, USA). The antibiotics, which contained fifteen sulfonamides (sulfacetamide, sulfadiazine, sulfamethylthiazole, sulfathiazole, sulfamerazine, sulfamethazine, sulfameter, sulfamonomethoxine, sulfachloropyridazine, sulfamethoxazole, sulfamethoxypyridazine, sulfadimethoxine, sulfisoxazole, sulfapyridine, and sulfadoxine), five quinolones (enrofloxacin, lomefloxacin, ciprofloxacin, ofloxacin and sarafloxacin) and three tetracyclines (chlortetracycline, doxycycline and oxytetracycline) were separated using C_18_ reversed-phase columns and subjected to mass spectrometer detections operated at selected reaction monitoring modes^11^.

### Heavy Metal Analysis

Six heavy metals (Mn, Co, Cu, Zn, Cd, and Pb) were selected for analysis that are frequently related to anthropogenic activities or are commonly used commercially. Heavy metal content was quantified by inductively coupled plasma-atomic emission spectroscopy^8^.

### Competitive experiments

Twenty sulfonamide-resistant *E*. *coli* were isolated from water samples in the Daliaohe and Liaohe River estuaries (ten sulfonamide-resistant *E*. *coli* from each estuary). More details are shown in SI Appendix. All resistant *E*. *coli* and non-resistant *E*. *coli* (ATCC25922) were incubated at 37 °C with shaking at 150 rpm/min for 15 h in liquid LB, and dilutions of each strain were prepared separately in 0.85% NaCl solution up to a final dilution of 0.5 McFarland standard. 20 μL of each dilution was added to reaction systems to evaluate the effects of nutrient status, salinity and sulfadimidine concentration on the growth of both antibiotic resistant and non-resistant *E*. *coli*. The reaction systems are shown in SI Appendix, Table S1. All reaction systems were incubated at 20 °C and with shaking at 150 rpm/min. Culture growth was then assessed by optical density at 600 nm using a microplate reader every two hours, and every eight hours after 10 hours. Lastly, the Gompertz model was used to calculate the lag and logarithmic growth phases of antibiotic resistant and non-resistant *E*. *coli* using the following equation^12,13^.

3 $$Y(t)=A+Cexp\{-exp[-B(t-M)]\}$$

Y(t): Y(t) = lgN, (N is the abundance of bacteria at t);

A: The logarithm of the abundance of bacteria at t → 0;

C: The difference between the logarithm of the highest abundance of bacteria at stationary phase and the logarithm of the abundance of bacteria at t → 0;

M: The time for maximum growth rate of bacterial culture (h);

B: Relative growth rate at M (h^−1^);

t: The time of bacterial culture.

### Statistical analyses

Statistical tests were performed using SPSS version 19.0 (SPSS) and MATLAB 2015. Statistical significance was defined at *p* < 0.05. One-way ANOVA was used to compare and determine the statistical significance between population means at 95% confidence intervals. Raw data was standardized using the min-max normalization method. Correlation analysis was used to calculate Pearson’s correlation coefficient (*r*) and statistical significance between dependent variables (concentration of sulfonamide-resistant *E*. *coli*, resistance levels) and independent variables (antibiotics, heavy metals, physicochemical parameters). The weight of each environmental factor was determined using gray relational analysis. Multivariable linear regression model analyses, paired-sample *t* tests, and ANOVA tests were used to infer the influence of environmental factors.

## Results and Discussion

### Distribution and sulfonamide-resistance levels of *E*. *coli* in estuaries

**Figure 1:**
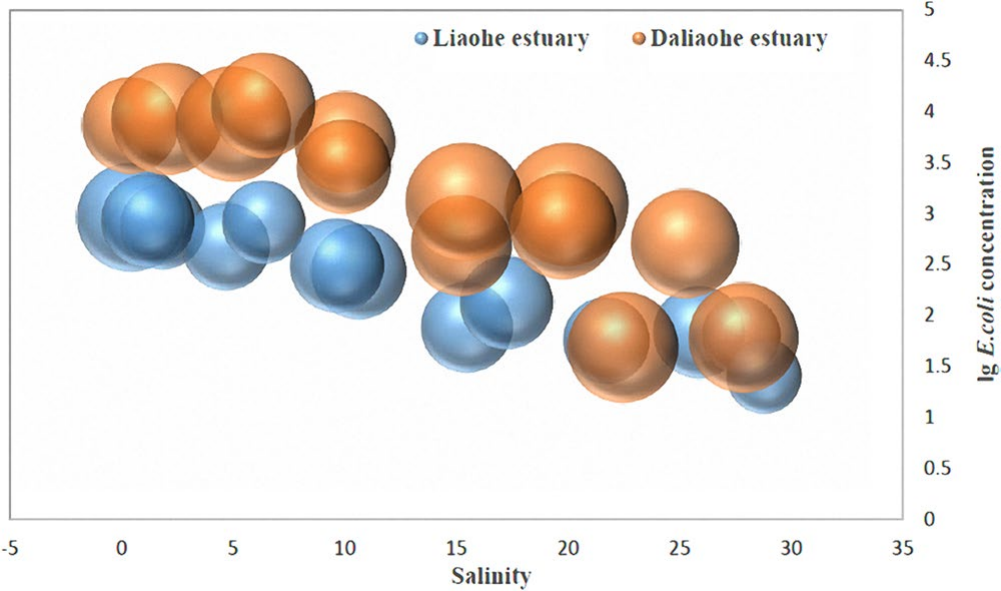
Distribution and sulfonamide-resistance levels of *E*. *coli* in the Daliaohe and Liaohe estuaries. Bubble size indicates relative level of antibiotic resistance.

*E*. *coli* contamination was determined in water samples from the Daliaohe and Liaohe River estuaries (Fig. 1) with abundances ranging from 25 CFU/100 ml to 11,400 CFU/100 ml in the estuaries. There was significant attenuation of *E*. *coli* abundances in the process of migration from the inland rivers to the ocean. The average attenuation rates of *E*. *coli* were 99.45% and 95.45% in the Daliaohe and Liaohe estuaries, respectively. The minimum abundance observed were dozens of strains per 100 mL, suggesting that estuaries play a natural attenuation role in inhibiting dispersal of antibiotic-resistant *E*. *coli* populations. The sulfonamide-resistance levels of *E*. *coli* were substantially lower than those previously reported from the Bernesga River in Northwest Spain^14^ and from the Dongjiang River in Michigan^15^. In contrast, the values observed here were consistent with previously reported direct cell counts from a river in Osun State^16^ and seawater from the South Fildes Peninsula^17^.

The dilution of seawater considerably affected the distribution of *E*. *coli* in the estuaries. The theoretical attenuation rate of *E*. *coli* abundances in estuaries was calculated with salinity as a reference value, and with dilution as the only considered factor in the migration process. The theoretical attenuation rates of *E*. *coli* abundances in the two estuaries were 69.48% and 71.67% in the Daliaohe and Liaohe estuaries, respectively, and the average contribution to the actual attenuation rate was 72.48%. On the other hand, decreased vertical transmission and loss of culturability were another major cause of *E*. *coli* dispersal attenuation in the estuaries studied here, as has been shown in similar environments^18,19^. The result indicates that the effective attenuation rate of sulfonamide-resistant *E*. *coli* abundances was close to 30% in the migration process from rivers to oceans. Moreover, the attenuation of resistance levels was strongly related to the attenuation of total *E*. *coli* abundances (*p* ≤ 0.05). Compared to the degree of attenuation of ARGs during estuary migration^8^, the attenuation of sulfonamide-resistant *E*. *coli* is more pronounced which indicate that differences in environmental adaptability between microbes might lead to the structure of ARGs changes in the estuary migration.

**Figure 2:**
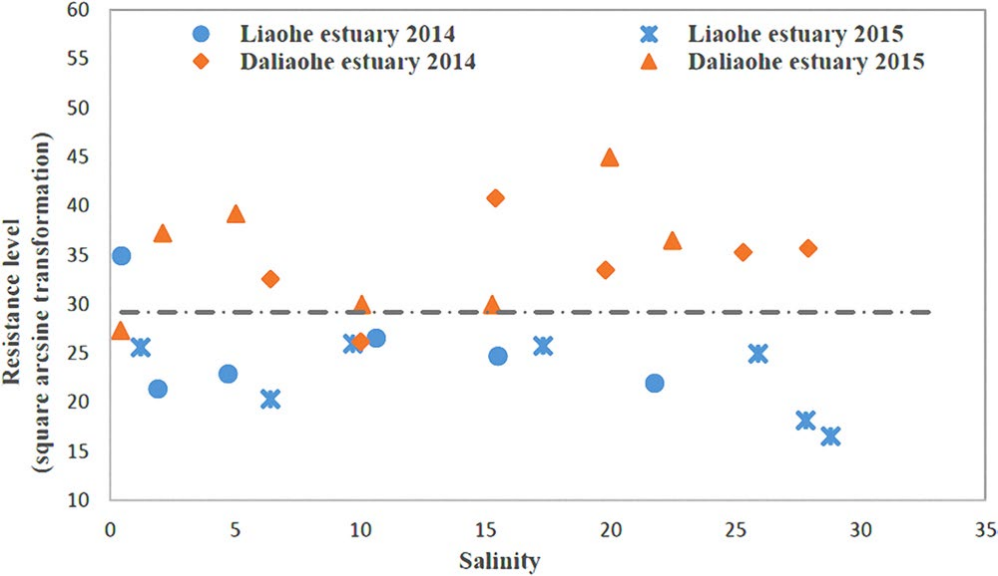
Sulfonamide-resistance levels of *E*. *coli* in the Daliaohe and Liaohe estuaries.

In order to compare the effect of anthropogenic influence on *E*. *coli* abundance and resistance levels, two estuaries were compared which experience differing levels of human-induced pollution. There were significant differences in the degree of pollution between the two regions and a trend towards diminishing pollution along the length of both estuaries. In the Daliaohe estuary, where the level of anthropogenic activity is relatively strong, both *E*. *coli* abundances and resistance levels were significantly higher than those in the Liaohe estuary (Fig. 2), where the level of anthropogenic activity is weaker. The average sulfonamide-resistant *E*. *coli* biomass in surface water samples of the Daliaohe and Liaohe estuaries were 804 CFU/100 ml and 52 CFU/100 ml in the summer, respectively, and 1,364 CFU/100 ml and 91 CFU/100 ml in the winter, respectively. Further, the abundance of sulfonamide-resistant *E*. *coli* in the dry season was higher than that in the wet season. Additionally, the average resistance level within the Liaohe estuary was only 51.49% of that in the Daliaohe estuary. The distribution pattern of sulfonamide-resistant *E*. *coli* was similar to *sul*-ARGs in the same region^8^. These results further suggested that the fate of antibiotic resistance pollution was susceptible to ambient pressures from anthropogenic activities.

### Complex Relationships among Environmental Factors and Sulfonamide -Resistant *E*. *coli*

**Figure 3:**
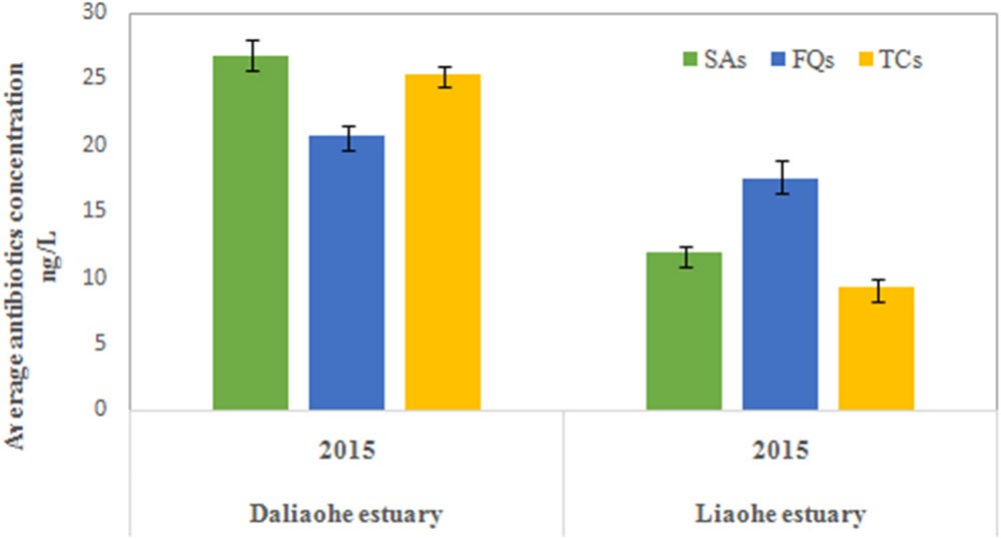
Average concentrations of antibiotics detected in water samples. Three different classes of antibiotics are shown: sulfonamides (SAs; green), quinolones (FQs; blue), and tetracyclines, (TCs; yellow). The average concentrations of antibiotics in 2014 could be seen in the article published in the 2015^8^.

**Figure 4:**
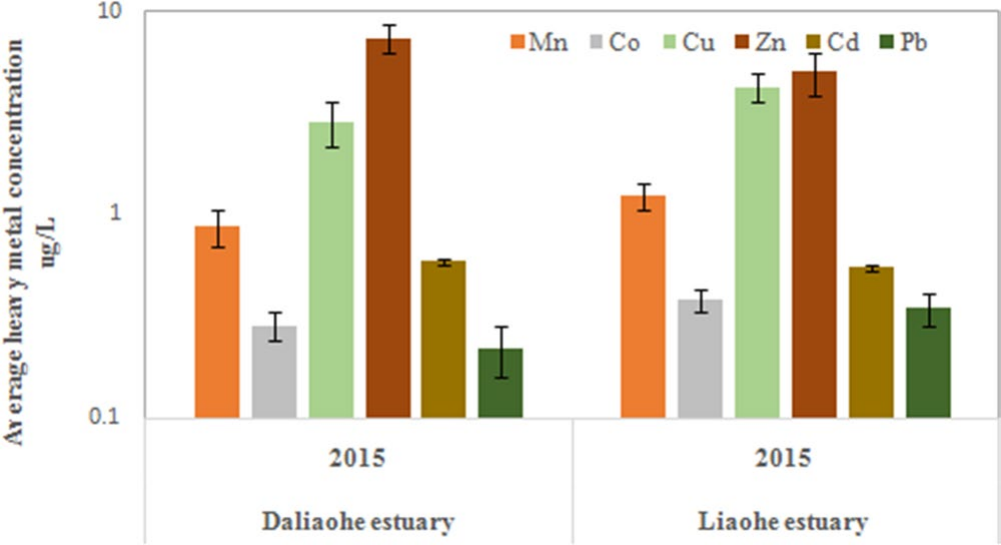
Average concentrations of heavy metals detected in water samples. The average concentrations of heavy metals in 2014 could be seen in the article published in the 2015^8^.

In order to further understand the distribution and migration of sulfonamide-resistant *E*. *coli* in estuaries and to explore the contribution of environmental factors in this process, we measured three types of antibiotics (Fig. 3), six heavy metals (Fig. 4) and three physicochemical parameters (Table 1). Antibiotics and heavy metals were widespread in the Daliaohe and Liaohe estuaries (Figs 3, 4). Despite the widespread prevalence of antibiotics and heavy metals in both estuaries, their concentrations were relatively low. For example, the average concentrations of total sulfonamide were 47.2 ng/L and 28.6 ng/L in water samples collected from the Daliaohe and Liaohe estuaries, respectively. Both of these sulfonamide ranges were lower than that of other inland regions, such as the Haihe River^20^ and the urban rivers in Beijing^21^. However, the degree of pollution was significantly different between the two estuaries. The concentrations of antibiotics and heavy metals were relatively higher in the Daliaohe estuary and were coincident with overall *E*. *coli* abundance differences between the two estuaries.

Significant correlations were not observed between sulfonamide-resistant *E*. *coli* abundances and sulfonamide antibiotic concentrations. This result indicates that continental-oceanic physicochemical and ecological interactions in estuary environments modulate the interactions between antibiotics and ARB. Different population dynamics of ARB caused by replicative and propagative biological characteristics result in differential correlations between ARB and antibiotics in inland aquatic environments and estuaries. Thus, it was assumed that the role of antibiotics in the transmission of resistance will be replaced or lessened due to antibiotic concentration decreases, the inability to transfer resistance over large spatial distances, and the increase of environmental change intensity. In this context, the primary transmission mechanism of antibiotic resistance should switch from “active communication” to “passive communication,” and a long-distance migration process likely becomes complex, involving multiple mechanisms and propagation pathways. Nevertheless, these results do not preclude that low concentration of antibiotics do not provide an effective selective pressure in estuary environments. It is more likely that the complex characteristics of natural environments result in complex relationships between ARB and antibiotics, making direct causative relationships between the two difficult to observe. Importantly, other environmental factors, besides antibiotic levels, may also affect the maintenance and proliferation of antibiotic resistance^22–24^.

**Figure 5:**
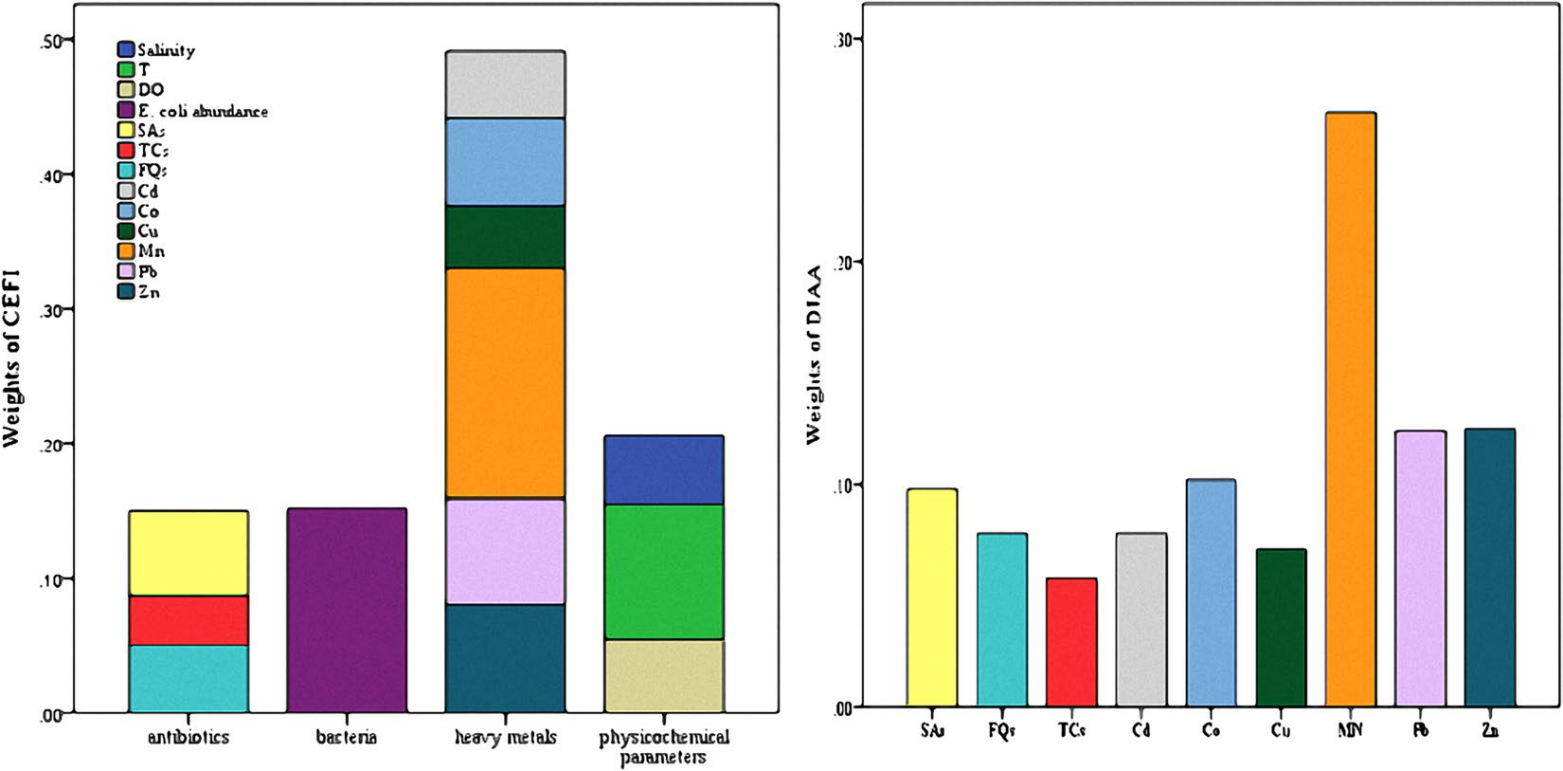
The weights of 13 environmental indicators in the comprehensive environmental factor index (CEFI) and the weight of each pollutant in the disturbance index of anthropogenic activities (DIAA).

In order to test the hypothesis that a complex set of environmental factors effect on sulfonamide-resistant *E*. *coli* migration dynamics, standardization of 13 environmental indicators was performed using the min-max normalization method according to the regional characteristics of target estuaries. The weights of each indicator in the comprehensive environmental factor index and the weight of each pollutant in the disturbance index of anthropogenic activities were determined using gray relational degree analysis (Fig. 5). The comprehensive environmental factors index (CEFI) and the disturbance index of anthropogenic activities (DIAA) were then calculated for each salinity transect in both estuaries based on the standardized data and indicator weights (Table 2). There was a significant positive correlation between the environmental factors index, the disturbance index of anthropogenic activities and the resistance levels of *E*. *coli* (Table 3). This correlation suggests that the overall effect of environmental factors on *E*. *coli* resistance and contamination is independent of spatial distribution and also indicates that the integrated effects of pollution in natural waters influences the distribution and migration of antibiotic resistant microorganisms. It is also important to note that environmental physicochemical parameters play an additional role in influencing the prevalence of ARB.

**Table 2:** The comprehensive environmental factors index (CEFI) and the disturbance index of anthropogenic activities (DIAA).

Region	2014			2015		
	Salinity (‰)	CEFI	DIAA	Salinity (‰)	CEFI	DIAA
Daliaohe River estuary	22.47	0.33	0.37	27.90	0.21	0.10
	19.97	0.30	0.30	25.30	0.22	0.11
	15.27	0.34	0.40	19.80	0.31	0.26
	10.05	0.41	0.42	15.40	0.27	0.19
	5.02	0.65	0.77	10.00	0.28	0.19
	2.09	0.58	0.65	6.40	0.38	0.17
	0.41	0.38	0.35	—	—	—
Liaohe River estuary	21.76	0.25	0.25	26.40	0.16	0.02
	15.50	0.26	0.28	27.10	0.30	0.24
	10.63	0.23	0.25	26.80	0.22	0.12
	4.70	0.24	0.27	28.10	0.27	0.22
	1.90	0.20	0.21	28.30	0.22	0.15
	0.44	0.22	0.24	28.30	0.19	0.09
	—	—	—	27.90	0.25	0.19

**Table 3:** Correlation analysis of CEFI, DIAA and the resistance levels of *E*. *coli* in estuary environment. In each cell, the top value indicates the Pearson correlation coefficient (*r*), and the bottom value in italics indicates the *p*-value. Bold values indicate statistical significance (*p* < 0.05).

	SAs	TCs	FQs	Mn	Co	Cu	Zn	Cd	Pb	CEFI	DIAA
Resistance levels *of E*. *coli*	0.25	**0**.**47**	0.23	0.37	0.26	0.10	**0**.**41**	−0.03	0.26	**0**.**47**	**0**.**43**
	0.21	**0**.**02**	0.26	0.06	0.20	0.63	**0**.**04**	0.88	0.19	**0**.**02**	**0**.**03**
Sulfonamide-resistant *E*. *coli* abundance	**0**.**39**	0.20	−0.09	**0**.**60**	**0**.**56**	0.24	**0**.**63**	0.03	**0**.**47**	**0**.**85**	**0**.**64**
	**0**.**05**	0.32	0.68	**0**.**00**	**0**.**00**	0.23	**0**.**00**	0.90	**0**.**02**	**0**.**00**	**0**.**00**

Multivariable linear regression model analysis was also used to assess the contribution of environmental indicators on the distribution and migration of antibiotic resistant *E*. *coli*. A strong multivariate linear fitting relationship was observed between antibiotic resistant *E*. *coli* abundances and environmental indicators (*R*^2^ = 0.986, *p ≤ *0.01). The contribution ratio of each indicator was calculated according to the partial regression coefficient for each. The abundance of *E*. *coli* was the most important factor influencing the abundance of antibiotic resistant strains, with a contribution ratio of 48.56%. The second-most influential factor was heavy metal content, with a total contribution ratio of 29.42% for the six heavy metals (Mn, Zn, Cu, Co, Cd, Pb). The contribution ratio of Zn was the highest among the metals (16.19%). The contribution ratio of the three measured physicochemical parameters on the distribution of antibiotic resistant *E*. *coli* was 14.64%. Finally, the contribution ratio of antibiotics was only 7.39%, with sulfonamides accounting for only 4.26%. These results indicate that the effect of antibiotics at low concentrations on the distribution and migration of antibiotic resistant strains is weak relative to other factors.

Unlike antibiotics, heavy metals are not subject to degradation and can consequently represent a long-standing, widespread and recalcitrant selection pressure which potentially contributes to the maintenance and spread of sulfonamide-resistant *E*. *coli* in estuary systems^8,25^. Higher heavy metal concentrations likely represent a strong selective pressure for bacteria, which may explain the higher influence contribution ratio. The strong correlations between ARB and heavy metals suggested that heavy metal contamination may be considered a major factor contributing to the migration of sulfonamide-resistant *E*. *coli*, particularly if antibiotic concentrations are very low (Table 3). These results are consistent with a number of reports that suggest that heavy metal contamination in natural environments can have an important role in the maintenance and propagation of antibiotic resistance^8,26,27^. Thus, heavy metals may be one of the dominant factors in off-setting the effect of sulfonamide-resistant *E*. *coli* vertical transmission in estuarine environments.

### Environmental Adaptability Simulation of Sulfonamide-resistant *E*. *coli*

To further understand the effect of estuarine physicochemical conditions and the role of antibiotics at low concentrations on ARB migration and competitive ability, 20 sulfonamide-resistant environmental strains were randomly selected to simulate the effects of salinity, nutritional and antibiotics stress. In the no-antibiotics conditions of the reference simulation system, the growth of antibiotic-resistant strains was less affected by salinity increases. When the salinity was increased from 5% to 35‰, the lag phase of sulfonamide-resistant *E*. *coli* increased by 39.25%, and the lag phase of non-resistant strains was prolonged by 50.87%. Additionally, the average generation time increase of antibiotic-resistant strains was lower than that of the non-resistant strains by 24.6%. The range of maximal biomass change was relatively small, but the reduction rate of the antibiotic-resistant bacteria was relatively lower than that of the non-resistant bacteria (Table 4). The decline of the nutritional status of the system also further affected the adaptability of resistant strains and non-resistant strains in response to environmental salinity stress (Table 4). When the nutritional status of the simulated system was decreased to 10%, the influence on antibiotic-resistant bacteria lag phase by salinity was only 59.56% of non-resistant bacteria. These results were also recapitulated in generation time and the maximal biomass between antibiotic-resistant and non-resistant *E*. *coli*. The deviation degree of lag phase and generation time between resistant strains and non-resistant strains was reversed from an initial +28.41% and +37.24 (100% nutrition, 0.5 salinity) to −9.33% and −4.06% (10% nutrition, 3.5 salinity). Overall, in the oligotrophic environment system in the sea, sulfonamide-resistant *E*. *coli* exhibit a stronger adaptability to the environment via increased migration ability that is correlated with salinity. Therefore, although the estuary could play a natural attenuator role in limiting the dispersal of antibiotic-resistance *Escherichia coli*, it could also improve the competitive advantage of antibiotic-resistant strains, thereby increasing the risk of ARB prevalence.

**Table 4:** Relative variation ratio in growth states of resistant strains and non-resistant strains. In each cell, the value indicates the relative variation ratio in growth states from 5‰ salinity increased to 35‰ salinity.

Nutrient status	Strain	lag phase	generation time	maximal biomass
100%	non-resistant	50.87%	85.07%	−2.71%
	resistant	39.25%	60.45%	−1.82%
20%	non-resistant	71.77%	110.73%	−2.13%
	resistant	53.69%	80.76%	−0.41%
10%	non-resistant	132.36%	130.05%	−1.90%
	resistant	78.84%	86.17%	−0.36%

When antibiotic stress was induced in the simulation system, the lag phases of sulfonamide-resistant *E*. *coli* and non-resistant strains were slightly increased, but there was no significant difference in the degree of influence at antibiotic concentrations in the hundreds of ng/L (Table 5). When antibiotic concentrations were greater than a few hundred µg/L, the lag phase of the antibiotic-resistant strain was significantly prolonged, and the effect of antibiotics on the non-resistant *E*. *coli* was more apparent. In addition, the influence of antibiotics on antibiotic-resistant strain generation time was similar as that of the effect on lag phase. The selective pressure of antibiotics (in the range of experimental concentrations) had no effect on the maximal biomass of strains in the poor nutrient and low salinity conditions (10% nutrition, 0.5 salinity). However, with increases of salinity and nutrient availability, antibiotic-resistant strains were gradually less successful. Antibiotic presence in the 10% nutrient simulation system lead to lag phase differences between resistant and non-resistant strains, which were reduced from 40.44% to 13.8% (350 ng/L antibiotics) and 1% (350 µg/L antibiotics). Additionally, the deviation in generation time change rate of resistant strains and non-resistant strains was reduced from 33.74% (without antibiotics) to 12.56% (350 ng/L) and −10.81% (350 µg/L). This effect was further amplified in the 100% nutrient status treatment. Thus, the selective pressure of antibiotics at low concentrations are amplified in certain circumstances, and this would enhance the competition of antibiotic-resistant strains and increase their occurrence and risk of antibiotic-resistance spread in natural environments. Nevertheless, because the proliferation of antibiotic-resistant strains is affected by many factors, the effect of antibiotics at low concentrations on ARB success in the simulation system may not be directly observed and the interaction between the two may even be dramatically reduced.

**Table 5:** Relative variation ratio in growth states of resistant strains and non-resistant strains. In each cell, the value indicates the relative variation ratio in growth states from 5‰ salinity increased to 35‰ salinity.

Nutrient status	antibiotic	Strains	lag phase	generation time	maximal biomass
100%	350 ng/L	non-resistant	31.73%	31.48%	0.00%
		resistant	52.32%	51.70%	−0.03%
	350 ug/L	non-resistant	36.66%	35.57%	0.00%
		resistant	51.01%	44.27%	0.36%
10%	350 ng/L	non-resistant	86.37%	75.84%	0.58%
		resistant	74.45%	66.25%	0.49%
	350 ug/L	non-resistant	80.72%	53.52%	1.58%
		resistant	79.92%	59.30%	1.17%

## Conclusions

The present study demonstrated that: 1. estuarine ecosystems play a natural attenuator role in the substantial reduction of sulfonamide-resistant *E*. *coli* abundances and in the migration process of ARB from rivers to oceans. However, there was no significant change in the resistance levels of *E*. *coli* during these migration processes. 2. The level of sulfonamide-resistant *E*. *coli* in estuarine water environments were significantly affected by environmental factors and the level of anthropogenic activities. However, the contribution of antibiotics towards the persistence of antibiotic-resistance levels is very small. 3. Lastly, simulation experiments showed that antibiotic-resistant *E*. *coli* were better adapted to withstand environmental changes. Nevertheless, the results indicate that low concentrations of antibiotics do not have the ability to increase the competitive advantage of sulfonamide-resistant *E*. *coli* in estuary environments.

## Electronic supplementary material

Supporting Information
