# Supplementary material for: The effect of environmental factors and migration dynamics on the prevalence of antibiotic-resistant Escherichia coli in estuary environments
Source: Sci Rep. 2018 Jan 26;8:1663. doi: 10.1038/s41598-018-20077-x (PMC5786026; doi:10.1038/s41598-018-20077-x)
Supplement: Supplementary file 1 — Supporting Information [file 41598_2018_20077_MOESM1_ESM.pdf]

Supporting Information Appendix

The effect of environmental factors and migration dynamics on the prevalence of antibiotic-resistant *Escherichia coli* in estuary environments

Guangshui Na[1*], Zihao Lu[1], Hui Gao[1], Linxiao Zhang[1, 2], Qianwei Li[1, 3], Ruijing Li[1], Fan Yang[1], Chuanlin Huo[1], Ziwei Yao[1]

[1] *Key Laboratory for Ecological Environment in Coastal Areas (SOA), National Marine Environmental Monitoring Center, Dalian 116023, China*

[2] *School of Biology Technology, Dalian Polytechnic University, Dalian 116034, China*

[3] *School of Marine Science, Shanghai Ocean University, Shanghai 201306, China*

Sampling Design

Liaohe River and Daliaohe River water systems are located between 40° 31' N to 45°17'N and 116° 54' E to125°32' E in northeastern China. Both water systems enter Liaodong Bay of Bohai Sea via Liaohe River and Daliaohe River estuaries, respectively. Nevertheless, the difference in pollution level between the two estuaries is obvious. Liaohe river estuary, as the national natural reserve, is under relatively few influences of concentrated anthropogenic activities. By contrast, the midstream and downstream areas of the Daliaohe River system are important industrial bases where oil, chemical, medicine, and steel factories in the northeast part of China are located and distributed in large industrial cities, such as Shenyang, Benxi, Anshan, and Yingkou. Approximately 2074 million tons of industrial and domestic wastewater is discharged annually into the Daliaohe River catchment (Guo et al., 2007). The ecological risk of the Daliaohe River estuary is relatively high. Therefore, comparative study between the Daliaohe and Liaohe river estuaries can provide extensive research opportunity regarding the fate of ARGs in the estuary environment under the effects of different anthropogenic activities. The sampling stations are shown in Fig. S1.

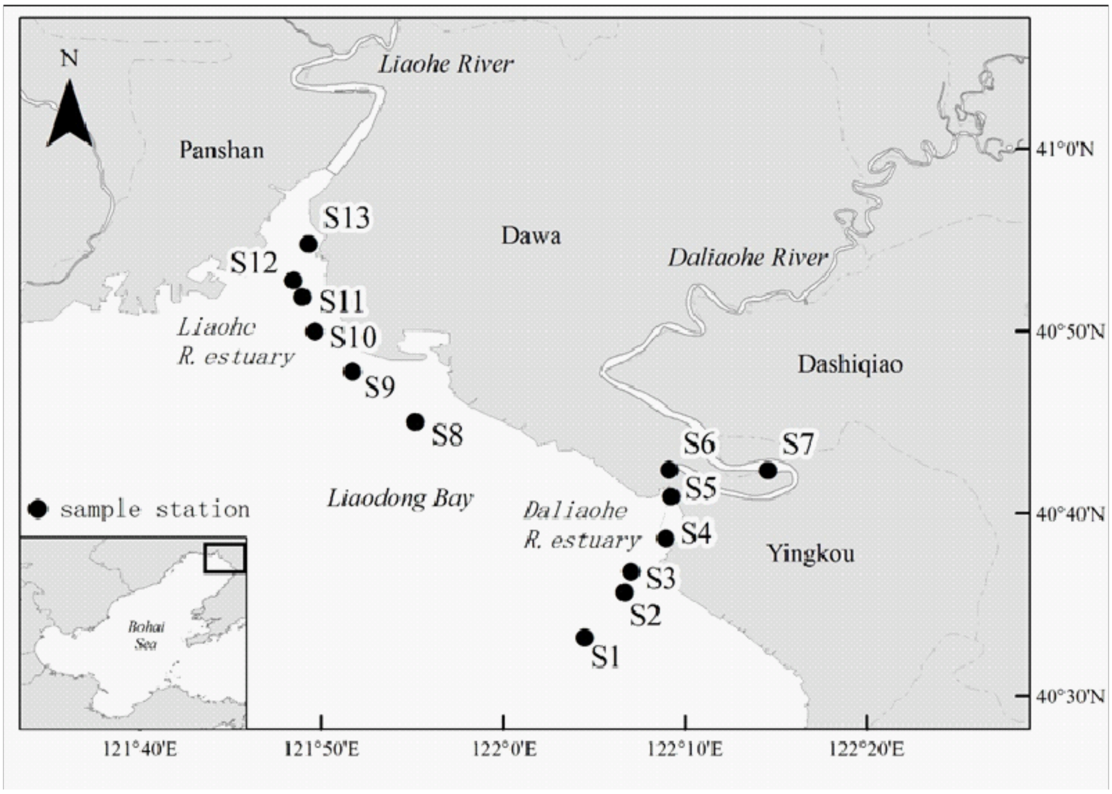

Figure S1. Location of the sampling sites (made by ArcGIS 10.3, standalone version)

Competitive experiments

Isolation of sulfonamide-resistant *E. coli*

We randomly picked off twenty colonies from the MI-R plates and used conventional methods for isolation and purification. One lactose fermented colony was further identified as *E. coli* by API 20E identification strips (bioMerieux, Marcyl'Etoile, France). Species identification of the isolates was performed by PCR that detects usp(A) gene, which is characteristic for *E. coli* strains (Chen and Griffiths, 1998). Antibiotic resistance of all the *E. coli* strains were identified *sul1* by PCR.

Table S1. The reaction systems

System	Sulfadimidine concentration	Nutrient status	Salinity
1	0	100%	0.5%
2	0	100%	3.5%
3	0	10%	0.5%
4	0	10%	3.5%
5	350ng/L	100%	0.5%
6	350ng/L	100%	3.5%
7	350ng/L	10%	0.5%
8	350ng/L	10%	3.5%
9	350μg/L	100%	0.5%
10	350μg/L	100%	3.5%
11	350μg/L	10%	0.5%
12	350μg/L	10%	3.5%

Equation of CEFI and DIAA

Comprehensive environmental factors index (CEFI)

$$CEFI_i = \sum_{j=1}^{m} w_j \times y_{ij}$$

w: weights of each indicator, y: standardization of environmental indicators

Disturbance index of anthropogenic activities (DIAA)

$$DIAA_i = \sum_{j=1}^{m} w'_j \times p_{ij}$$

w': weights of each pollutant, p: standardization of pollutants

References

Guo, W. *et al.* Distribution of polycyclic aromatic hydrocarbons in water, suspended particulate matter and sediment from Daliao River watershed, China. *Chemosphere* **68**, 93-104 (2007).

Chen, J. & Griffiths, M. W. PCR differentiation of Escherichia coli from other gram-negative bacteria using primers derived from the nucleotide sequences flanking the gene encoding the universal stress protein. *Letters in Applied Microbiology* **27**, 369-371 (1998).
